# Treatment of allergic bronchopulmonary mycosis: Experience of 55 patients with 124 relapses—A descriptive study

**DOI:** 10.1002/ccr3.2305

**Published:** 2019-09-18

**Authors:** Takashi Ishiguro, Ayako Kojima, Naomi Takata, Noboru Takayanagi

**Affiliations:** ^1^ Department of Respiratory Medicine Saitama Cardiovascular and Respiratory Center Saitama Japan; ^2^ Department of Radiology Saitama Cardiovascular and Respiratory Center Saitama Japan

**Keywords:** allergic bronchopulmonary aspergillosis, allergic bronchopulmonary mycosis, corticosteroid, relapse, treatment

## Abstract

There is no established consensus for the treatment of allergic bronchopulmonary mycosis (ABPM) on its diagnosis or at relapse. We reviewed our experience with patients with ABPM, which showed that although systemic corticosteroids are effective in ABPM, and other treatment options can also be selected.

## Graphical abstract

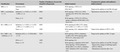

## 1 INTRODUCTION

We conducted a retrospective study of 55 patients with allergic bronchopulmonary mycosis and median follow‐up period of 2311 days from diagnosis to review their treatment results. As a result, systemic corticosteroids are effective, but other treatment options can also be selected at diagnosis or at relapse of the disease.

Allergic bronchopulmonary mycosis (ABPM) is an allergic disease characterized by eosinophilic inflammation that causes the development of mucoid impaction of the bronchi (MIB) and central bronchiectasis. Systemic corticosteroids (SCSs) are a mainstay of its treatment, and a limited number of treatment regimens have been suggested on its diagnosis.^1^ Recently, the efficacy of antifungal agents has been reported,^2^ and further studies are needed to determine appropriate treatment regimens on the diagnosis of ABPM. In addition, patients with ABPM unfortunately experience relapses during their clinical courses, but there have been no studies investigating treatment options and their efficacy in treating relapses. Thus, the aim of the present study was to assess whether SCSs should always be administered on diagnosis and at each relapse, and whether other treatment options can also be selected.

## 2 PATIENTS AND METHODS

**Figure 1:**
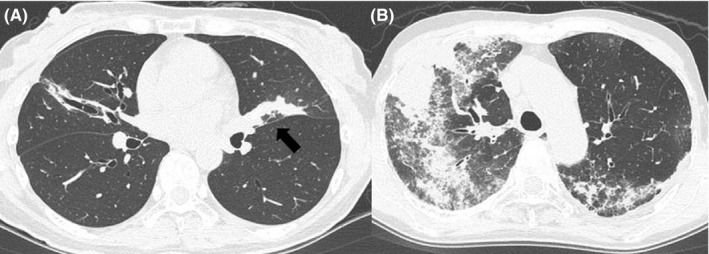
Chest computed tomography in patients with allergic bronchopulmonary mycosis. Chest computed tomography showed shadows filling the bronchial lumen (A, arrow) and pulmonary consolidations (B)

We conducted a retrospective study of 55 patients who were diagnosed as having ABPM by established diagnostic criteria^3^, ^4^, ^5^ from March 1993 to November 2017 at Saitama Cardiovascular and Respiratory Center. Dyspnea with episodic wheezes and/or rhonchi was regarded as symptoms of bronchial asthma [BA]. Development of cough and sputum alone was not regarded as BA symptoms because such symptoms were nonspecific. We classified BA symptoms as severe or nonsevere based on the Japanese Asthma Prevention and Management Guideline,^6^ in which severe BA symptoms are defined as difficulty in breathing in the supine position or on effort, or more severe symptoms. The existence of findings of shadows filling the bronchi, which was regarded as MIB (Figure 1A), and consolidation (Figure 1B) on chest computed tomography (CT) images was reviewed. Conditions of ABPM on diagnosis and on relapses were classified based on the existence of BA symptoms and shadows of MIB and consolidation on CT. When patients in a stable condition developed BA symptoms and/or new shadows on CT, they were considered to have suffered a relapse. Patterns of relapse were also classified in the same manner as at diagnosis. Drugs administered until the time of diagnosis and relapses (pretreatment) and drugs that were added on or changes of treatments from the pretreatment regimen (treatment) were reviewed. Both pretreatment and treatment drugs were classified into three categories: SCSs, inhalation including inhaled corticosteroid (ICS) and β‐stimulants, and antifungal agents. When patients had been treated by SCSs and were newly administered itraconazole (ITCZ), SCSs were regarded as pretreatment, and ITCZ was regarded as treatment. When doses of inhalation drugs were increased or another inhalation drug was added (eg, pretreatment was an ICS and a β‐stimulant was newly added), these changes were also regarded as treatment. The treatment effect was evaluated 6‐8 weeks after starting each treatment. In patients with BA symptoms, when wheezes and dyspnea improved, we regarded the treatment as “effective.” In patients with new shadows, we regarded treatment as effective when both the symptoms and the shadows improved. In contrast, if these changes were not found, we regarded the treatment as “ineffective.” In ineffective cases, the subsequent treatment and its efficacy were also evaluated in the same manner. The treatment duration of SCSs was defined as “short duration” when SCSs were stopped within 2 weeks and as “tapering” when patients received SCSs for more than 2 weeks, and SCSs were then tapered.

Data are expressed as the mean value ± standard deviation (SD) unless otherwise specified. This study was approved by the ethics committee of Saitama Cardiovascular and Respiratory Center.

## 3 RESULTS

### 3.1 Patient demographics

Patients aged 61.8 ± 13.5 years old were included in our study (Table 1). Twenty (36.4%) were men, and a smoking history was found in 23 (41.8%) of the 55 patients. The median follow‐up period (range) from diagnosis was 2311 days (313‐7359 days). Underlying diseases in our patients included allergic diseases in 8 (14.5%), asthma in 34 (61.8%), old pulmonary tuberculosis in 5 (9.1%), diabetes mellitus in 4 (7.2%), and hypertension in 15 (27.3%). Laboratory data obtained on diagnosis included a mean white blood cell count of 8050 ± 2359/mm^3^, median (range) eosinophil count of 700 (0‐10 300)/mm^3^, and median (range) serum immunoglobulin E (IgE) level of 1453 (9‐11 061) IU/mL. The mean values of vital capacity and forced expiratory volume in 1 second on diagnosis were 2.7 ± 0.90 L and 1.80 ± 0.56 L, respectively (Table 1). Chest CT showed MIB and central bronchiectasis in all patients. Fifty‐four patients underwent bronchoscopy, and pathological findings of eosinophilic mucous plugs containing fungal hyphae, which were compatible with ABPM,^7^ were confirmed in 34 patients. During their follow‐up, 38 patients developed 124 relapses, whereas 17 did not experience any relapses.

**Table 1:** Patient characteristics

Age, y	61.8 ± 13.5
Male sex (%)	20 (36.4)
Smoking history, yes (%)	23 (41.8)
Dust exposure, yes (%)	2 (3.6)
Follow‐up duration, median (range)	2,311 (313‐7359)
Underlying diseases (%)	
Allergic diseases	8 (14.5)
Bronchial asthma	34 (61.8)
COPD	0 (0)
Interstitial lung diseases	0 (0)
Old tuberculosis	5 (9.1)
Diabetes mellitus	4 (7.2)
Hypertension	15 (27.3)
Respiratory failure (%)	3 (5.5)
Pulmonary function test results	
Vital capacity (L)	2.7 ± 0.90
FEV_1_ (L)	1.80 ± 0.56
Laboratory data	
WBC, mean ± SD (/mm^3^)	8050 ± 2359
Eosinophils, median (range; /mm^3^)	700 (0‐10 300)
Serum IgE, median (range; IU/mL)	1453 (9‐11 061)
Chest computed tomography	
Central bronchiectasis, yes	55
Mucoid impaction, yes	55

Note

Allergic diseases include pollinosis, allergic rhinitis, and atopic dermatitis.

Abbreviations: COPD, chronic obstructive pulmonary diseases; FEV_1_, forced expiratory volume in 1 s; WBC, white blood cell; SD, standard deviation; IgE, immunoglobulin E.

Causative fungi were *Aspergillus* sp in 36, *Schizophyllum commune* in 8, *Penicillium* sp in 3, *Pycnoporus sanguineus* in 1, *Perenniporia tephropora* in 1, and unknown in 9 (the number of causative fungi included cases in which multiple fungi were causative).

### 3.2 Drugs administered for pretreatment and treatment at relapse in each category

Systemic corticosteroids included prednisolone (PSL), methylprednisolone, and hydrocortisone. Inhaled corticosteroids included fluticasone, ciclesonide, budesonide, and inhaled β‐stimulants included long‐acting β‐agonists of formoterol, salmeterol, and vilanterol, and the short‐acting β‐agonist of salbutamol. The antifungal agent administered in our patients was ITCZ.

### 3.3 Clinicoradiological patterns on diagnosis and efficacy of each initial treatment

Conditions of the patients with ABPM on diagnosis could be classified into four patterns: MIB in 2, BA + MIB in 8, MIB + consolidation in 12, and BA + MIB+consolidation in 33. Patients' conditions and treatment effects on diagnosis are listed in Table 2. Of our patients, 25 received initial treatment that did not include SCSs, of whom 19 improved. Treatment effects (number of effective episodes/total episodes) on diagnosis regardless of patients' conditions were as follows: SCSs tapering ± inhalation, 20/22 (90.9%); ITCZ ± inhalation, 11/13 (84.6%); inhalation monotherapy, 7/8 (87.5%); SCSs tapering + ITCZ±inhalation 8/8 (100%); and observation, 2/4 (50%).

**Table 2:** Initial treatment on diagnosis and its efficacy on each pattern of the patients' conditions

Classification	Pretreatment	Number of incidences of severe BA attack/fever/hypoxemia	Initial treatment	Treatment for patients with ineffective initial treatment
MIB, n = 2	Inhalation (ICS)	0/0/0	SCSs tapering + ITCZ (1/1)	
	None		ITCZ (0/1)^a^	^a^Improved by addition of oral PSL 60 mg/d
MIB + consolidation, n = 12	Inhalation, n = 6 (ICS, n = 4; ICS/LABA, n = 2)	0/0/0	SCSs tapering + ITCZ (1/1), SCSs tapering (2/2); ITCZ (2/2); observation (0/1)^b^	^b^Spontaneous remission after 5 mo
	None, n = 6	0/0/0	SCSs tapering + ITCZ (1/1), SCSs tapering ± inhalation (ICS/LABA) (2/2), ITCZ (3/3)	
BA + MIB, n = 8	Inhalation, n = 5 (ICS, n = 3; ICS/LABA, n = 1; LABA, n = 1)	0/0/0	SCSs tapering±(LABA) (3/3), inhalation (LABA, n = 1; ICS, n = 1) (2/2)	
	None, n = 3	1/0/0	SCSs tapering (1/2)^c^, inhalation (ICS) (1/1)	^c^Improved by addition of ITCZ + ICS
BA + MIB + consolidation, n = 33	Inhalation (ICS, n = 5; ICS/LABA, n = 8)	5/2/4	SCSs tapering +ITCZ ± inhalation (LABA) (4/4), SCSs tapering (6/6); ITCZ (1/1), observation (1/2)^d^	^d^Improved by addition of PSL 15 mg/d
	SCSs, n = 1	1/0/0	SCSs tapering (1/1)	
	None, n = 19	3/1/1	SCSs tapering ± inhalation (ICS) (5/6)^e^, SCSs tapering + ITCZ+inhalation (ICS/LABA) (1/1), ITCZ ± inhalation (5/6)^f^, inhalation (4/5)^g^, observation (1/1)	^e^Improved by addition of oral PSL 20 mg/d, ^f^Improved by change of antifungal agents (ITCZ to VRCZ), ^g^Spontaneous remission after 3 mo

Abbreviations: BA, bronchial asthma; EP, eosinophilic pneumonia; ICS, inhaled corticosteroid; LABA, long‐acting beta agonist; MIB, mucoid impaction of the bronchi; PSL, prednisolone; SCSs, systemic corticosteroids; VRCZ, voriconazole.

Systemic corticosteroids were effective in all but two patients regardless of their clinicoradiological patterns: one patient with BA + MIB and another with BA + MIB+consolidation. The daily median dose (range) of SCSs (PSL) administered for treatment was 20 (10‐40) mg. All patients with at least one symptom of severe BA, fever, and hypoxemia received SCSs. Antifungal agents with or without other drugs were also effective in most patients.

Inhalation monotherapy was effective in all three patients with BA + MIB and in four of the five patients with a BA + MIB+consolidation pattern. Patients who received inhalation monotherapy did not receive SCSs. Patients without severe BA symptoms, fever, and hypoxemia did not always need SCSs but improved with the addition of ITCZ, introduction or strengthening of inhalation therapy, and observation.

### 3.4 Patterns on relapse and efficacy of each treatment

**Figure 2:**
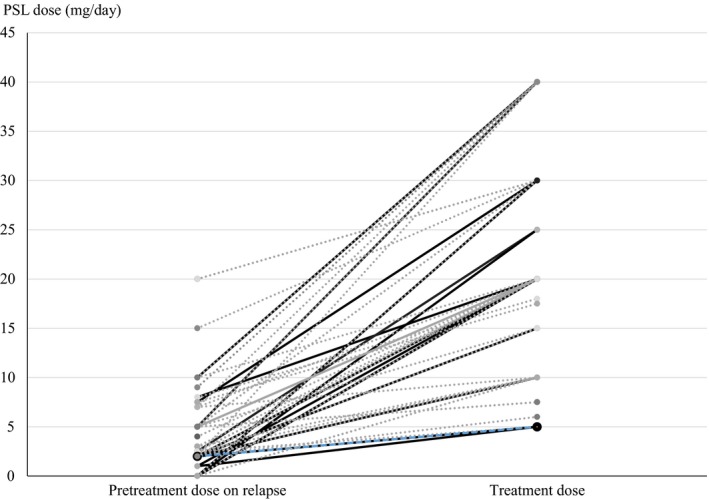
Pretreatment and treatment doses of corticosteroid. Solid lines indicate corticosteroid doses in patients who received treatment other than corticosteroids. Dotted lines indicate corticosteroid doses in patients who received corticosteroids alone on relapse. The blue dotted line indicates the corticosteroid dose in a patient with failure of corticosteroid therapy. Corticosteroid doses are expressed as those of prednisolone

Conditions of the patients with ABPM and the 124 relapses in total could be classified into six patterns: BA in 26, MIB in 53, consolidation in 5, BA + MIB in 2, MIB + consolidation in 36, and BA + MIB+consolidation in 2. Patients' conditions on relapse and treatment effects are listed in Tables 3 and 4. All patients with fever had consolidation on CT. Treatment effects on relapse regardless of the patients' conditions were as follows: inhalation, 9/10 (90.0%); SCSs ± inhalation, 53/54 (98.1%); ITCZ ± inhalation, 13/16 (81.3%); SCSs + ITCZ ± inhalation, 10/10 (100%); and observation, 22/36 (61.1%). Inhalation monotherapy was often selected in patients with airway diseases (BA and MIB) but was not selected in patients with consolidation. Inhalation monotherapy was effective in eight of nine patients with relapse patterns of BA or MIB who had been treated with ≤2 mg/d of PSL or without SCSs. Among the patients with relapse patterns of BA or MIB, 12 patients received SCSs for a short duration (<2 weeks) that resulted in improvement in all 12. ITCZ ± inhalation therapy was selected in patients with MIB and BA + MIB + consolidation, whereas ITCZ was not administered to patients with BA alone. As for the efficacy of ITCZ on patients with SCSs pretreatment, three patients pretreated with PSL of 2, 5, and 15 mg/d, respectively, improved with additional ITCZ, whereas another patient pretreated with PSL 2.5 mg/d did not improve with additional ITCZ. The daily dose of administered SCSs (regardless of the combination of other treatments) is shown in Figure 2. Only one patient (with pretreatment of PSL 2 mg/d and ICS) with MIB + consolidation did not improve with SCSs administration (increase in PSL dose from 2 to 5 mg/d) but then subsequently improved with additional ITCZ. Patients who were treated with observation at their relapse had mild symptoms and/or mild shadows (small band‐like shadows or pulmonary consolidations of limited size) on chest CT and were without fever or hypoxemia. Twenty‐two of the 35 patients who were treated with observation at their relapse spontaneously improved within 8 weeks, whereas the other 13 patients improved within 8 weeks following additional SCSs, ITCZ, inhalation, and further observation. In patients with consolidation, treatment effect (number of effective cases/total number of cases) was obtained with SCSs ± inhalation in 17/18 (94.4%), ITCZ ± inhalation in 9/9 (100%), SCSs + ITCZ ± inhalation in 7/7 (100%), and observation in 6/9 (66.7%) (Table 2).

**Table 3:** Pretreatments and treatment on relapse of ABPM with BA, MIB, and BA plus MIB

Classification	Pretreatment	Pretreatment PSL dose (mg/d)	Number of incidences of severe BA attack/fever/hypoxemia	Treatment (introduction or changes from pretreatment)	Following treatment for patients with ineffective treatment
BA, n = 26	None, n = 2	–	0/0/0	SCSs short duration + inhalation (added ICS) (1/1), observation (1/1)	
	Inhalation, n = 6 (ICS/LABA, n = 4; ICS, n = 2)	–	1/0/0	SCSs tapering (1/1), inhalation (3/3) (increased dose of ICS/LABA, n = 2, added SABA, n = 1), observation (2/2)	
	SCSs + ITCZ±inhalation, n = 7	2 (n = 5), 5, 10	0/0/0	SCSs short duration (4/4), SCSs tapering (1/1), inhalation (added ICS/LABA [2/2])^a^	
	SCSs ± inhalation, n = 11	2 (n = 3), 3, 5, 7 (n = 2), 8, 9, 10, 15	1/0/1	SCSs short duration ± inhalation (added ICS) (6/6), SCSs tapering + inhalation (added LABA, n = 1, increased dose of ICS/LABA, n = 1) (2/2), inhalation (added LABA) (1/1), observation (1/2)^b^	bImproved by increased dose of PSL + ITCZ
MIB, n = 53	ITCZ ± inhalation (ICS, n = 2; ICS/LABA, n = 3) n = 7	–	0/0/0	Inhalation (increased dose of ICS, added ICS) (1/2)^c^, observation (3/5)^d^	^c^Spontaneous improvement after 6 mo, ^d^Spontaneous improvement after 4 mo in 2 patients
	Inhalation (ICS, n = 2, ICS/LABA, n = 4) n = 6	–	0/0/0	SCSs tapering + ITCZ (1/1), SCSs short duration (1/1), ITCZ (0/1)^e^, inhalation (added LABA) (1/1), observation (0/2)^f^	^e^Spontaneous improvement after 4 mo, ^f^Improved by SCSs tapering
	None, n = 4	–	0/0/0	ITCZ + inhalation (added ICS/LABA) (2/2), ITCZ (1/2)^g^	^g^Improved by SCSs tapering
	SCSs + ITCZ±inhalation (ICS, n = 2, LABA, n = 1, ICS/LABA, n = 7), n = 21	2 (n = 4), 2.5 (n = 6), 3, 5 (n = 5), 9, 10, 20, 30	0/0/0	SCSs tapering ( 11/11), observation (6/10)^h^	^h^Spontaneous improvement after 3 and 4 mo, Spontaneous improvement after 4 mo
	SCSs ± inhalation (ICS, n = 4, LABA, n = 1, ICS/LABA, n = 7), n = 15	2, 2.5 (n = 2) 3, 5 (n = 3), 7 (n = 2), 8, 11, 15, 22.5	0/0/0	SCSs tapering (7/7), SCSs tapering + ITCZ (1/1), ITCZ (1/2)^i^, observation (3/5)^j^	^i^Improved by SCSs tapering, ^j^Spontaneous improvement after 4 mo
BA + MIB, n = 2	SCSs + inhalation (ICS/LABA), n = 1	2 (n = 1)	0/0/0	SCSs tapering + ITCZ+inhalation (added LABA) (1/1)	
	ITCZ + inhalation (ICS), n = 1	–	0/0/0	SCSs tapering + inhalation (added LABA) (1/1)	

Abbreviations: ABPM, allergic bronchopulmonary mycosis; BA, bronchial asthma; EP, eosinophilic pneumonia; ICS, inhaled corticosteroid; ITCZ, itraconazole; LABA, long‐acting beta agonist; MIB, mucoid impaction of the bronchi; PSL, prednisolone; SABA, short‐acting beta agonist; SCSs, systemic corticosteroids.

a Both patients had received PSL 2 mg/d.

b Patient had received PSL 2 mg/d.

**Table 4:** Pretreatments and treatments on relapse of ABPM with consolidation, MIB plus consolidation, and BA plus MIB plus consolidation

Classification	Pretreatment	PSL dose (mg/d)^a^	Number of incidences of severe BA attack/fever/hypoxemia	Introduction or change of treatment (number of effective cases/total cases)	Outcomes of patients with ineffective therapy
Consolidation, n = 5	SCSs + ITCZ, n = 1	2.5	0/0/0	SCSs tapering (1/1)	
	SCSs + inhalation (ICS), n = 2	8, 15	0/0/0	SCSs tapering (1/1)	
				Observation (1/1)	
	ITCZ + inhalation (ICS), n = 1		0/0/0	Observation (1/1)	
	Inhalation alone (ICS/LABA), n = 1		0/0/0	SCSs tapering (1/1)	
MIB + consolidation, n = 36	SCSs + ITCZ±inhalation (ICS, n = 1; ICS/LABA, n = 1), n = 4	2, 2.5, 4, 5	0/0/0	SCSs tapering (3/3), observation (1/1)	
	SCSs ± inhalation (LABA, n = 2, ICS, n = 4, ICS/LABA, n = 6), n = 16	1 (n = 2), 2 (n = 5), 3 (n = 2), 5 (n = 4), 8 (n = 2), 15	0/1/4	SCSs tapering ± inhalation (added ICS) (8/9)^a^, SCSs tapering + ITCZ±inhalation (4/4), ITCZ (2/2), observation (1/1)	^a^Improved by additional ITCZ
	ITCZ ± inhalation (ICS/LABA, n = 4), n = 5		0/1/0	SCSs tapering (1/1), SCSs short duration + ITCZ (increased dose) (1/1), ITCZ (increased dose)+inhalation (added ICS/LABA) (1/1), observation (1/2)^b^	^b^Spontaneous improvement after 3 mo
	Inhalation (ICS, n = 1, ICS/LABA, n = 4), n = 5		0/0/0	SCSs tapering + ITCZ (1/1), ITCZ (3/3), observation (0/1)	
	None, n = 6		0/0/0	SCSs tapering (1/1), ITCZ (3/3), observation (1/2)^c^	^c^Spontaneous improvement after 6 mo
BA + MIB +consolidation, n = 2	Inhalation (ICS/LABA), n = 1		0/0/0	SCSs short duration (1/1)	
	None, n = 2		0/0/0	SCSs tapering + ITCZ+inhalation (added ICS/LABA) (1/1)	

Abbreviations: ABPM, allergic bronchopulmonary mycosis; BA, bronchial asthma; ICS, inhaled corticosteroid; ITCZ, itraconazole; LABA, long‐acting beta agonist; MIB, mucoid impaction of the bronchi; PSL, prednisolone; SCSs, systemic corticosteroids.

a Number of patients is 1 when not indicated.

### 3.5 Treatments the patients were receiving at their final follow‐up

Treatments that the patients were receiving at their final follow‐up visit included antifungal monotherapy in 2 (3.6%), SCSs + antifungal therapy ± inhalation in 4 (7.3%), antifungal therapy + inhalation in 3 (5.5%), SCSs ± inhalation in 28 (50.9%), inhalation in 11 (20.0%), observation without any drugs in 6 (10.9%), and mepolizumab monotherapy in 1 (1.8%). The ABPM of all patients was stable, and none of them showed any symptoms or radiologic findings of ABPM at final follow‐up. The patient who was receiving mepolizumab monotherapy at final follow‐up had been initially treated with SCSs and inhalation, but BA symptoms, peripheral blood eosinophilia, and abnormal shadows had been refractory to treatment. Thus, mepolizumab had been started, which replaced the SCSs and inhalation therapy and maintained the patient's condition without further relapses. One patient with ABPM treated with PSL of 5 mg/d developed chronic progressive pulmonary aspergillosis during his clinical course and died 7 years after the diagnosis of ABPM. Another patient died of acute myocardial infarction 11 years after the diagnosis of ABPM.

### 3.6 Serum IgE and peripheral eosinophil counts at final follow‐up

A serum IgE value > 1000 IU/mL was found in 15 patients, 500 < IgE ≤ 1000 IU/mL in 9, and <500 IU/mL in 25 on final follow‐up. The number of peripheral blood eosinophils present at final follow‐up was ≥500/mm^3^ in seven patients.

## 4 DISCUSSION

We investigated the patterns of patients' conditions in terms of symptoms and radiologic patterns of ABPM at diagnosis and at relapse and treatment effects for each condition. Based on pathologic analyses of resected lung specimens of ABPM,^8^ the leading pathologic and pathogenic feature of ABPM is reported to be MIB. Peripheral consolidation develops as a result of the inhalation of allergic fungal antigen that causes eosinophilic pulmonary inflammation,^8^ and we regarded MIB and peripheral changes (consolidation) as the main components of the radiologic patterns of ABPM. Asthma is also known as a predisposing condition of ABPM, and thus, we classified our patients' conditions by combination of these components. As a result, conditions could be classified into four patterns on diagnosis and into six patterns at relapse.

Systemic corticosteroids are the mainstay of ABPA therapy in the initial treatment of ABPM. A previous study reported that treatment effects do not differ between a median dose (0.5 mg/kg/d) and high dose (0.75 mg/kg/d) of PSL, but the former is better than the latter because of its fewer adverse effects.^9^ An established guideline also recommended the addition of antifungal agents to SCSs to reduce the dose of SCSs^10^; however, American allergologists have been reported to select certain treatment options other than SCSs with or without antifungals,^11^ indicating that physicians need treatment options that do not include SCSs. Previously, we reported that infectious events occur more frequently than expected in patients with ABPM, and there is concern that SCSs can increase or worsen these events.^12^ In fact, one male patient with ABPM in our hospital developed chronic progressive pulmonary aspergillosis during his clinical course. Other patients who have developed invasive pulmonary aspergillosis and chronic progressive pulmonary aspergillosis under SCSs have also been reported.^13^, ^14^ Nineteen of our 25 patients who did not receive SCSs as an initial treatment improved, and we found that each treatment option without SCSs was effective at various different frequencies. For example, the efficacy of antifungal agents has been reported,^15^, ^16^, ^17^ and Agarwal et al found no significant differences in the characteristics of responders to ITCZ vs nonresponders.^2^ Identifying responders will allow patients with some risk factors to avoid SCSs, for example, patients with diabetes mellitus, osteoporosis, and active infectious diseases.

Another treatment option is inhalation therapy. Although inhalation is not considered to be useful for ABPM 18 and a previous report indicated that radiologic worsening cannot be suppressed by ICS regardless of patients' symptoms,^19^ our study found some patients who had not been treated with SCSs to be effectively treated by inhalation. One opinion is that the effects of additive inhalation may be minor in patients treated with PSL above 10 mg/d,^1^ but our results indicated that inhalation may also be effective in patients treated with a low dose of SCSs. Inhalation was effective in our patients with BA and MIB, and some patients with consolidation also improved with inhalation. Consolidation complicating ABPM is generally regarded as eosinophilic pneumonia (EP) although we cannot rule out the possibility that consolidations peripherally distributed to MIB include atelectasis. Inhalation therapy is not generally used in idiopathic acute or chronic EP, but our results indicated its efficacy in the EP that accompanies ABPM. We think that inhalation therapy improved the airway lesions (MIB), which resulted in the improvement of EP. These results suggest that physicians should not exclude ICS as a treatment option, especially when the patient is pretreated with a low dose of SCSs or without SCSs.

To our knowledge, treatment for the relapse of ABPM has not been fully investigated. Our study classified patients' conditions at relapse into six patterns. SCSs with tapering of their doses with or without other drugs were effective regardless of other pretreatments, indicating that SCSs are also a steadily effective treatment for relapses. However, all patients with relapse patterns of BA or MIB who received short‐duration SCSs also improved, indicating that such patients may not always require long‐term treatment with SCSs. Inhalation was also effective in eight of nine patients with relapse patterns of BA or MIB. As an initial treatment, inhalation may be effective in patients with consolidation, but it was not selected in patients with consolidation at their relapse. All of our patients with hypoxemia or fever had consolidation and received SCSs; thus, the efficacy of inhalation for consolidation at relapse is unknown, and SCSs are recommended at relapse in such patients. To summarize, our results showed that SCSs, inhalation, and antifungal agents can be included as treatments of choice at relapse. Although the efficacy of SCSs is good, they do not always need to be started or have their doses increased at relapse. Categories of drugs that are not administered just at relapse can be added, or the dosage of a drug that has already been prescribed can be increased, or another drug in the same category can be selected. Future studies should clarify the characteristics of patients who can be effectively treated with each treatment option.

Biologics have developed in the areas of severe asthma and allergic diseases, and other cases showing the efficacy of mepolizumab^20^ and omalizumab^21^ have also been reported. Mepolizumab replaced SCSs in one of our patients who at final follow‐up has remained well controlled and has developed neither BA symptoms nor new shadows on chest imaging. Further studies are needed to clarify the efficacy of biologics for ABPM, but biologics could potentially change the treatment order of ABPM.

Our study also included patients who spontaneously improved by observation within 6‐8 weeks. Patients treated with observation at relapse had mild symptoms without fever or hypoxemia and mild radiologic findings. Some patients who showed no spontaneous improvement within 6 to 8 weeks received oral corticosteroids or ITCZ and then showed improvement. Other patients continued to be observed after 6‐8 weeks, and they subsequently improved within 3 to 6 months. Prolonged inflammation of ABPM is considered to lead to irreversible structural damage, and we do not know how long the inflammation can be observed and left untreated. If several months cannot be tolerated, then observation is not an adequate treatment.

Our study has several limitations. First, it was conducted retrospectively at a single institution. The number of patients was small, and we could not fully evaluate the results statistically. Second, although the serum IgE value has been reported to be a marker of ABPM relapse,^22^, ^23^, ^24^ we did not always measure it. However, we performed CT on all patients, and all relapses were well evaluated by that modality. Third, we did not perform drug monitoring of antifungal agents, whose efficacy may have been difficult to evaluate. Fourth, patients with BA frequently take other anti‐inflammatory drugs to control their BA (eg, leukotriene receptor antagonist or theophylline), but their effects were not evaluated because their efficacy in treating ABPM has not been appreciated. Fifth, doses of ICSs were not evaluated in detail. Finally, the efficacy of each treatment option may not derive solely from its effects alone because the treatment drugs can interact with other drugs^25^ that have already been administered.

## 5 CONCLUSION

At both diagnosis and relapse, at least two treatment options other than SCSs are available, including antifungals and inhalation therapy. Biologics may also be included as an additional option. Future studies should clarify how to predict the efficacy of each treatment option and devise an elaborate treatment algorithm on initial diagnosis and at relapse.

## CONFLICT OF INTEREST

Authors do not have any other conflicts of interest to declare.

## AUTHOR CONTRIBUTIONS

TI: is the guarantor of the paper, taking responsibility for the integrity of the work as a whole, from inception to published article. AK and Noboru T: aggregated the data, created the tables, and helped draft the discussion of the manuscript. Naomi T: reviewed the CT findings of each of the patients throughout their clinical course.

## References

[1] Agarwal R . Allergic bronchopulmonary aspergillosis. Chest. 2009;135:805‐826. 19265090 10.1378/chest.08-2586

[2] Agarwal R , Dhooria S , Singh Sehgal I , et al. A randomized trial of itraconazole vs prednisolone in acute‐stage allergic bronchopulmonary aspergillosis complicating asthma. Chest. 2018;153:656‐664. 29331473 10.1016/j.chest.2018.01.005

[3] Agarwal R , Chakrabarti A , Shah A , et al. ABPA complicating asthma ISHAM working group. Allergic bronchopulmonary aspergillosis: review of literature and proposal of new diagnostic and classification criteria. Clin Exp Allergy. 2013;43:850‐873. 23889240 10.1111/cea.12141

[4] Rosenberg M , Patterson R , Mintzer R , Cooper BJ , Roberts M , Harris KE . Clinical and immunologic criteria for the diagnosis of allergic bronchopulmonary aspergillosis. Ann Intern Med. 1977;86:405‐414. 848802 10.7326/0003-4819-86-4-405

[5] Ishiguro T , Takayanagi N , Uozumi R , et al. Diagnostic criteria that can most accurately differentiate allergic bronchopulmonary mycosis from other eosinophilic lung diseases: a retrospective, single‐center study. Respir Investig. 2016;54:264‐271. 10.1016/j.resinv.2016.01.004 27424826

[6] Japanese Society of Allergology . Asthma prevention and management guideline 2018. Tokyo: Kyowa Kikaku; 2018.

[7] Bosken CH , Myers JL , Greenberger PA , Katzenstein AL . Pathologic features of allergic bronchopulmonary aspergillosis. Am J Surg Pathol. 1988;12:216‐222. 3344888 10.1097/00000478-198803000-00007

[8] Hebisawa A , Tamura A , Kurashima A , et al. Pathologic reconsideration on allergic bronchopulmonary aspergillosis and mycosis. Nihon Kokyuki Gakkai Zasshi. 1998;36:330‐337. (In Japanese). 9691645

[9] Agarwal R , Aggarwal AN , Dhooria S , et al. A randomized trial of glucocorticoids in acute‐stage allergic bronchopulmonary aspergillosis complicating asthma. Eur Respir J. 2016;47:385‐387. 26585431 10.1183/13993003.01475-2015

[10] Walsh T , Anaissie E , Denning D , et al. Infectious diseases society of america. Treatment of aspergillosis; clinical practice guidelines of the infectious diseases society of America. Clin Infect Dis. 2008;46:327‐360. 18177225 10.1086/525258

[11] Greenberger PA , Bush RK , Demain JG , Luong A , Slavin RG , Knutsen AP . Allergic bronchopulmonary aspergillosis. J Allergy Clin Immunol Pract. 2014;2:703‐708. 25439360 10.1016/j.jaip.2014.08.007 PMC4306287

[12] Ishiguro T , Takayanagi N , Baba Y , Takaku Y , Kagiyama N , Sugita Y . Pulmonary nontuberculous mycobacteriosis and chronic lower respiratory tract infections in patients with allergic bronchopulmonary mycosis without cystic fibrosis. Intern Med. 2016;55:1067‐1070. 27150856 10.2169/internalmedicine.55.5561

[13] Maturu VN , Agarwal R . Acute invasive pulmonary aspergillosis complicating allergic bronchopulmonary aspergillosis: case report and systematic review. Mycopathologica. 2015;180:209‐215. 10.1007/s11046-015-9907-0 26045286

[14] Lowes D , Chishimba L , Greaves M , Denning DW . Development of chronic pulmonary aspergillosis in adult asthmatics with ABPA. Respir Med. 2015;109:1509‐1515. 26507434 10.1016/j.rmed.2015.09.007

[15] Salez F , Brichet A , Desurmont S , Grosbois J‐M , Wallaert B , Tonnel A‐B . Effects of itraconazole therapy in allergic bronchopulmonary aspergillosis. Chest. 1999;116:1665‐1668. 10593792 10.1378/chest.116.6.1665

[16] Denning DW , Van Wye JE , Lewiston NJ , Stevens DA . Adjunctive therapy of allergic bronchopulmonary aspergillosis with itraconazole. Chest. 1991;100:813‐819. 1653680 10.1378/chest.100.3.813

[17] Stevens DA , Schwartz HJ , Lee JY , et al. A randomized trial of itraconazole in allergic bronchopulmonary aspergillosis. N Engl J Med. 2000;342:756‐762. 10717010 10.1056/NEJM200003163421102

[18] Agarwal R , Khan A , Aggarwal AN , Saikia B , Gupta D , Chakrabarti A . Role of inhaled corticosteroids in the management of serological allergic bronchopulmonary aspergillosis (ABPA). Intern Med. 2011;50:855‐860. 21498933 10.2169/internalmedicine.50.4665

[19] Inhaled beclomethasone dipropionate in allergic bronchopulmonary aspergillosis. Report to the research committee of the British thoracic association. Br J Dis Chest. 1979;73:349‐356. 400108 10.1016/s0007-0971(79)80172-2

[20] Terashima T , Shinozaki T , Iwami E , Nakajima T , Matsuzaki T . A case of allergic bronchopulmonary aspergillosis successfully treated with mepolizumab. BMC Pulm Med. 2018;18:53. 29587693 10.1186/s12890-018-0617-5 PMC5870493

[21] van der Ent CK , Hoekstra H , Rijkers GT . Successful treatment of allergic bronchopulmonary aspergillosis with recombinant anti‐IgE antibody. Thorax. 2007;62:276‐277. 17329558 10.1136/thx.2004.035519 PMC2117163

[22] Patterson R , Greenberger PA , Halwig JM , Liotta JL , Roberts M . Allergic bronchopulmonary aspergillosis: natural history and classification of early disease by serologic and roentgenographic studies. Arch Intern Med. 1986;146:916‐918. 3516103 10.1001/archinte.146.5.916

[23] Vlahakis NE , Aksamit TR . Diagnosis and treatment of allergic bronchopulmonary aspergillosis. Mayo Clin Proc. 2001;76:930‐938. 11560305 10.4065/76.9.930

[24] Agarwal R , Gupta D , Aggarwal AN , Behera D , Jindal SK . Allergic bronchopulmonary aspergillosis lessons from 126 patients attending a chest clinic in north India. Chest. 2006;130:442‐448. 16899843 10.1378/chest.130.2.442

[25] Skov M , Main KM , Sillesen IB , Müller J , Koch C , Lanng S . Iatrogenic adrenal insufficiency as a side‐effect of combined treatment of itraconazole and budesonide. Eur Respir J. 2002;20:127‐133. 12166560 10.1183/09031936.02.00248002

